# Seed migration after transperineal interstitial prostate brachytherapy by using loose seeds: Japanese prostate cancer outcome study of permanent iodine-125 seed implantation (J-POPS) multi-institutional cohort study

**DOI:** 10.1186/s13014-015-0532-3

**Published:** 2015-11-14

**Authors:** Masahiro Nakano, Atsunori Yorozu, Shiro Saito, Akitomo Sugawara, Shinichiroh Maruo, Shinsuke Kojima, Takashi Kikuchi, Masanori Fukushima, Takushi Dokiya, Hidetoshi Yamanaka

**Affiliations:** Department of Urology, Gifu University Graduate School of Medicine, 1-1Yanagito, Gifu-shi, Gifu, 501-1194 Japan; Department of Radiation Oncology, National Hospital Organization, Tokyo Medical Center, 2-5-1 Higashigaoka, Meguro-ku, Tokyo, 152-8902 Japan; Department of Urology, National Hospital Organization Tokyo Medical Center, 2-5-1 Higashigaoka, Meguro-ku, Tokyo, 152-8902 Japan; Department of Radiation Oncology, Tokai University, Hachioji Hospital, 1838 Ishikawa-machi, Hachioji-shi Tokyo, 192-0032 Japan; Translational Research Informatics center, 1-5-4 Minatojima-minamimachi Chuo-ku, Kobe, Hyogo 650-0047 Japan; Department of Radiology, Kyoundo Hospital, 1-8 Kandasurugadai, Chiyoda-ku, Tokyo, 101-0062 Japan; Institute of Preventive Medicine, Kurosawa Hospital, 187 Yanakamachi, Takasaki-shi, Gunma 370-1203 Japan

**Keywords:** Prostate cancer, Brachytherapy, Seed migration

## Abstract

**Background:**

The incidence and associated factors of loose seed migration were investigated in cohort 1 of the Japanese Prostate Cancer Outcome Study of Permanent Iodine-125 Seed Implantation (J-POPS).

**Methods:**

The study subjects were 2160 patients, consisting of 1641 patients who underwent permanent iodine-125 seed implantation (PI) and 519 patients who underwent PI combined with external beam radiation therapy (PI + EBRT). The presence or absence of seed migration to the chest and abdominal/pelvic region was determined.

**Results:**

Seed migration was observed in 22.7 % of PI group patients and 18.1 % of PI + EBRT group patients (*p* = 0.0276). Migration to the lungs and abdominal/pelvic region was observed in 14.6 % and 11.1 % of the patients in the PI group, and 11.2 % and 8.5 % of the patients in the PI + EBRT group, respectively. In the PI group, the number of implanted seeds was associated with the seed migration incidence. Neither the PI nor the PI + EBRT group showed any difference in the volume of the prostate receiving 100 % of the prescribed dose (V100 [%]) or the minimal dose received by 90 % of the prostate volume (D90 [Gy]) between the patients with and without seed migration.

**Conclusions:**

This prospective cohort study investigating the largest number of past cases showed no difference in D90 (Gy) or V100 (%) between seed migration or the absence thereof in both the PI group and PI + EBRT group.

**Trial registration:**

ClinicalTrials.gov: NCT00534196

## Background

Transperineal permanent iodine-125 seed implantation (PI) for prostate cancer is widely accepted as a standard procedure for treating early-stage prostate cancer [1, 2]. It has shown favorable treatment outcomes [3, 4]. In Japan, treatment with iodine-125 (I-125) was introduced in July 2003 and is widely accepted [5, 6].

Seed migration is a well-known adverse event associated with PI. The seed migration incidence is reported to be 1.7–69.4 % [7–9]. There is a well-developed venous plexus around the prostate, and seeds are implanted near or within the veins. Thus, the seeds enter blood vessels and are transported by blood flow, which is considered to be the mechanism causing seed migration [10–13]. Various locations have been reported as sites of seed migration, including the lungs, abdomen, pelvic cavity, heart [14], vertebral venous plexus [15], kidneys [16], liver [17], and testicular veins [18]. As for the seed migration incidence, there are many reports of studies concerning only migration to the lungs, whereas only a few reports describe studies including migration to sites other than the lungs [9].

There is no report that comparatively investigates the incidence of seed migration by dividing between a PI group and PI combined with external beam radiation therapy (PI + EBRT) group. Past reports also had a small sample size of 20 to 495 cases that were investigated for seed migration, and nearly all reports came from only a single facility. [8–13, 19–26].

Our study is a nationwide multi-institutional cohort study called the Japanese Prostate Cancer Outcome Study of Permanent Iodine-125 Seed Implantation (J-POPS; NCT00534196) [5]. We report the results of our investigation of the incidence of seed migration with loose seeds by using J-POPS data, and of the factors associated with the occurrence of migration.

The present study is the world's first reported prospective cohort study on I-125 loose seed migration, and investigates a record number of cases.

## Methods

### J-POPS study

The J-POPS is a prospective cohort study evaluating the clinical significance of PI in Japan [5]. All enrolled patients had been diagnosed with histologically proven and clinically localized prostate cancer. The indications and contraindications for treatment were determined according to the American Brachytherapy Society (ABS) recommendations [27]. Of 2354 patients enrolled in cohort 1 from 46 facilities between July 2005 and June 2007, we included 2160 patients, excluding 2 duplicate enrollments, 12 previously untreated patients, 11 ineligible patients, 34 patients without data on the presence or absence of seed migration, and 145 patients who did not undergo chest radiography. Moreover, patients in whom seeds were shed into the seminal vesicles or bladder were not included in the group of patients with seed migration.

This study was approved by the foundation for Biochemical Research and Innovation. The ethical review committee of the Translational Research Informatics Center (TRI); the Institutional Review Boards of all participating facilities approved this study.

The treatment technique and recommended radiation field and dosimetry have been described previously [5]. Briefly, according to the American Brachytherapy Society recommendations, preplanning was undertaken using transrectal ultrasonography. The clinical target volume (CTV) was determined from gross target volume that was defined as the prostate volume visualized on ultrasonography with an added treatment margin of 3-5 mm in all directions, except for <2 mm in the posterior direction. The CTV for PI and PI + EBRT was same. In PI cases, the prescribed radiation doses were set so that the values of the minimal dose received by 90 % of the prostate volume (D90) would be 144 Gy for the CTV. In the cases of PI + EBRT, the prescribed radiation doses were set so that the D90 values would be 100–110 Gy for the CTV, and the prescribed radiation doses in EBRT were set at 40–50 Gy, which was recommended to be delivered in 20–25 fractions. Post-planning was undertaken using CT scanning approximately one month after treatment. For pre- and post-planning, Interplant version 3.0 to 3.4, manufactured by CMS, or VariSeed version 7.0 to 7.1, manufactured by VARIAN, software was used. I-125 seeds were loose seeds, and OncoSeed (Nihon Mediphysics^©^) or Brachy source (Bird^©^) was used and implanted using a Mick applicator (Mick TP 200 Mick Radio-Nuclear Instruments) in all patients [5].

### Methods to evaluate seed migration

Seed migration was checked during a period from the following day to three months after PI. The presence or absence of migration to the lungs was checked by chest radiography (with a posterior-anterior [PA] view alone or PA and lateral [PA + lateral] views), and the presence or absence of migration to the abdomen or pelvis was checked with plain abdominal radiography except for only one institution (35 cases) checked with plain abdominopelvic CT. Patients were divided into the PI group (1641 patients) and the PI + EBRT group (519 patients) for assessment.

### Toxicity scoring and follow-up schedule

In order to grade adverse events associated with the rectum and urination conditions, the National Cancer Institute Common Terminology Criteria for Adverse Events (NCI-CTCAE) version 3.0 were used. Adverse events were assessed at 3, 12, 24, and 36 months after PI, and the highest grade during this period was used for analysis [5].

### Statistical analyses

Wilcoxon signed-rank test and chi-square analysis were used to determine the strength of the relationships between seed migration and clinical treatment parameters. A 0.05 level of statistical significance and confidence intervals with 95 % probability were set. Statistical analyses were performed using SAS statistical software (version 9.3, SAS Institute Inc., Cary, NS, USA). All statistical analyses were performed at the TRI [5].

### Ethical considerations

This study was reviewed by the ethical review committee of each participating facility and conducted after written consent was obtained from patients [5].

## Results

### Methods and timeline of checking of seed migration

The methods used to evaluate seed migration were chest radiography with the PA view plus plain abdominal radiography in 1283 patients (59.4 %), chest radiography with the PA + lateral views plus plain abdominal radiography in 834 patients (38.6 %), and chest radiography with the PA view plus abdominal CT in 43 patients (2.0 %). The timeline of checking for seed migration was the day after implantation in 244 patients (11.3 %), one month after implantation in 1276 patients (59.0 %), and three months after implantation in 640 patients (29.6 %).

### Incidence and sites of seed migration

Table 1 shows the patient characteristics stratified by the treatment strategies and the presence or absence of seed migration.

**Table 1:** Patient characteristics stratified according to the seed migration and treatment methods

	PI			PI + EBRT		
	Migration (−)	Migration (+)	p*	Migration (−)	Migration (+)	p*
	(*n* = 1269)	(*n* = 372)		(*n* = 425)	(*n* = 94)	
Age: mean (median)	68.0 (69.0)	67.4 (68.0)	0.1257	69.0 (70.0)	68.4 (69.0)	0.3808
Range	45-89	51-84		52-88	53-82	
PSA (ng/mL): mean (median)	7.1 (6.4)	7.2 (6.4)	0.9354	10.7 (9.3)	10.8 (10.4)	0.7603
Range	1.6-41.8	2.0-33.0		1.9-42.0	3.6-27.2	
BMI: mean (median)	23.6 (23.4)	23.7 (23.7)	0.1524	23.5 (23.4)	23.5 (23.3)	0.8588
Range	14.7-32.8	17.9-32.1		15.5-35.7	17.3-29.7	
Prostate volume (mL)						
Mean (median)	26.2 (25.5)	27.5 (26.5)	0.0046	24.1 (23.4)	25.1 (24.1)	0.2927
Range	7.3-71.0	10.4-56.9		7.0-61.9	11.0-60.9	
Number of seeds						
Mean (median)	72.6 (72.0)	77.4 (75.0)	<0.0001	51.5 (50.0)	53.2 (50.5)	0.1044
Range	34-120	26-120		25-85	35-85	
Number of migrated seeds						
Mean (median)	-	1.6 (1.0)		-	1.5 (1.0)	
Range	-	1-9		-	1-5	
Number of needles						
Mean (median)	22.9 (22.0)	23.3 (23.0)	0.1913	18.1 (17.0)	18.4 (18.0)	0.3142
Range	10-45	12-41		9-36	11-32	
Planning methods						
Preplanning	320	60	0.1483	59	11	0.7385
Preplanning + intraoperative	949	312		366	83	
Loading methods						
Peripheral loading	229	56	0.3308	35	12	0.2408
Modified peripheral loading	1033	313		384	82	
Modified uniform loading	7	3		6	-	
Androgen deprivation therapy						
Yes	699	231	0.0174	117	22	0.4429
No	569	141		308	72	
Unknown	1	-		-	-	
Clinical T stage						
T1	965	302	0.1329	252	48	0.2662
T2	295	70		161	42	
T3	2	-		12	4	
Unknown	7	-		-	-	
Gleason score						
6 or less	873	262	0.5907	62	23	0.0028
7	384	108		312	52	
8 or more	10	1		51	19	
Unknown	2	1		-	-	
Risk classification (D’Amico)						
Low	733	220	0.9139	9	4	0.0419
Intermediate	505	146		334	63	
High	17	4		82	27	
Unknown	14	2		-	-	
Period of checking for migration						
Day 1	164	42	0.4247	29	9	0.3806
1-3 months	1105	330		396	85	

*: Wilcoxon signed test for qualitative variables, Fisher’s Exact for quantitative variables

*PI* = permanent iodine-125 seed implantation; *PI+EBRT* = PI combined with external beam radiation therapy; *PSA* = prostate specific antigen; *BMI* = body mass index

Out of all 2160 patients, 466 (21.6 %) showed seed migration. It was observed in 22.7 % (372/1641) of patients in the PI group and 18.1 % (94/519) of patients in the PI + EBRT group. Seed migration incidence in the PI group was significantly higher than that in the PI + EBRT group (*p* = 0.0276). Among patients with seed migration, the mean of migrating seeds per patient was 1.6 seeds (range, 1–9 seeds) in the PI group and 1.5 seeds (range, 1–5 seeds) in the PI + EBRT group. The number of migrating seeds was two seeds or less in 87.6 % in PI group and 86.2 % in PI + EBRT group. Migration to the lungs was observed in 14.6 % (240/1641) of PI group patients and 11.2 % (58/519) of PI + EBRT group patients. Moreover, migration to sites other than the lungs was observed in 11.1 % (182/1641) and 8.5 % (44/519) in the PI and PI + EBRT groups, respectively (Table 2).

**Table 2:** Location and number of migrated seeds stratified according to treatment methods

	PI (%)	PI + EBRT (%)	p*
Migration			
Yes	372 (22.7)	94 (18.1)	0.0276
No	1269 (77.3)	425 (81.9)	
Site of migration			
Lung	184 (49.5)	47 (50.0)	
Lung + pelvis	48 (12.9)	10 (10.6)	
Lung + abdomen	5 (1.3)	1 (1.1)	
Lung + abdomen + pelvis	2 (0.5)	-	
Lung + others	1 (0.3)	-	
Pelvis	115 (30.9)	30 (31.9)	
Abdomen	8 (2.2)	2 (2.1)	
Abdomen + pelvis	1 (0.3)	-	
Spinal venous plexus	-	1 (1.1)	
Thigh	2 (0.5)	-	
Unknown	6 (1.6)	3 (3.2)	
Number of migrated seeds			
1	242 (65.1)	60 (65.2)	
2	84 (22.6)	21 (22.8)	
3	25 (6.7)	8 (8.7)	
4	9 (2.4)	2 (2.2)	
5	5 (1.3)	1 (1.1)	
6	3 (0.8)	-	
7	3 (0.8)	-	
8	-	-	
9	1 (0.3)	-	

*: Chi square test

*PI* = permanent iodine-125 seed implantation; *PI+EBRT* = PI combined with external beam radiation therapy

The proportions of migrating seeds out of the total number of implanted seeds were 0.49 % in the PI group (120822 seeds implanted in total) and 0.53 % in the PI + EBRT group (26887 seeds implanted in total).

Within the PI group, the prostate volume (*p* = 0.0046) and the number of implanted seeds (*p* < 0.0001) were significantly larger in patients with seed migration than in those without seed migration (Table 1).

The Japanese national guideline recommends that total administered radionuclide activity be kept below 1300 MBq. Therefore, we had to reduce the prostate volume to around 30 cc with neoadjuvant androgen deprivation therapy (ADT) in case of PI monotherapy [6]. We presume that those may have been the reasons why the rate of patients treated with ADT was higher in the PI group than in the PI + EBRT group (Table 1).

When patients with seed migration were divided into those with seed migration confirmed on Day 1 and those with seed migration confirmed during a period from one to three months after implantation to assess the seed migration incidence, no significant difference was observed in either the PI or PI + EBRT group (Table 1).

### Impact on radiation doses

**Fig. 1:**
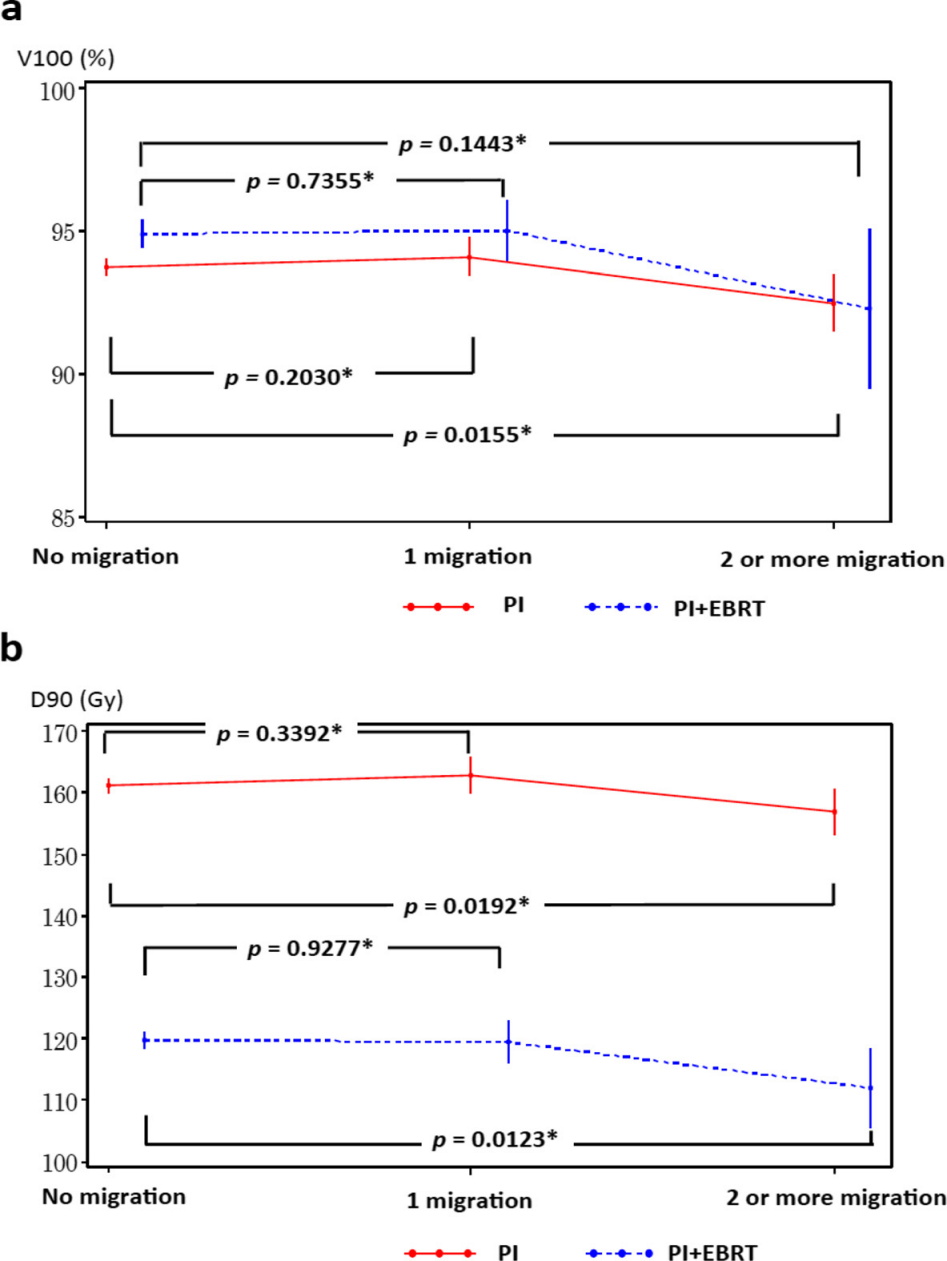
Dosimetric comparison of migration numbers. **a**: D90 (Gy), **b**: V100 (%) D90 (Gy) = minimal dose received by 90 % of the prostate volume; V100 (%) = volume of the prostate receiving 100 % of the prescribed dose; PI = permanent iodine-125 seed implantation; PI + EBRT = PI combined with external beam radiation therapy *: Wlicoxon singed-rank test

In the PI group, no significant difference in the volume of the prostate receiving 100 % of the prescribed dose (V100 [%]) and D90 (Gy) values was observed between patients with and without seed migration (*p* = 0.7332 and *p* = 0.5866, respectively), whereas the values of volume of the prostate receiving 150 % of the prescribed dose (V150 [%]), the minimal dose received by 90 % of the urethral volume (UD90 [Gy]) and the minimal dose received by 5 % of the urethral volume (UD5 [Gy]) were significantly lower in patients with seed migration (*p* = 0.0184, *p* = 0.0258 and *p* = 0.0371, respectively). On the other hand, the PI + EBRT group showed no significant difference in these prescribed doses between patients with and without seed migration (Table 3). When the D90 (Gy) and V100 (%) values were analyzed by dividing patients in both the PI and PI + EBRT groups into three groups without seed migration, with one migrating seed, and with two or more migrating seeds, the values were significantly lower in the group with two or more migrating seeds compared to the group without seed migration (Fig. 1).

**Table 3:** Dosimetric comparison of migration or no migration

	PI			PI + EBRT		
	Migration (−)	Migration (+)	p*	Migration (−)	Migration (+)	p*
	(*n* = 1269)	(*n* = 372)		(*n* = 425)	(*n* = 94)	
D90 (Gy)						
Mean (median)	161.1 (160.9)	160.7 (159.8)	0.5866	119.9 (120.5)	117.7 (119.2)	0.2627
Range	78.0-231.9	86.4-223.7		60.2-191.6	74.3-161.4	
V100 (%)						
Mean (median)	93.7 (94.8)	93.5 (94.9)	0.7332	94.9 (96.2)	94.1 (95.9)	0.2491
Range	63.6-100.0	56.3-100.0		56.5-100.0	68.3-99.8	
V150 (%)						
Mean (median)	62.6 (63.5)	60.7 (60.7)	0.0184	63.6 (64.6)	60.9 (61.1)	0.0731
Range	20.8-94.6	18.4-91.6		16.3-90.7	30.3-87.0	
UD90 (Gy)						
Mean (median)	141.7 (142.5)	138.2 (136.1)	0.0258	110.3 (111.1)	106.9 (104.7)	0.1019
Range	7.9-336.5	15.7-255.3		40.0-180.0	43.5-170.4	
UD5 (Gy)						
Mean (median)	228.6 (225.2)	222.4 (218.7)	0.0371	169.0 (161.5)	162.6 (160.2)	0.1570
Range	119.0-427.0	125.9-338.2		97.6-338.4	102.0-251.6	

*: Wilcoxon signed test for qualitative variable

*D90* = minimal dose received by 90 % of the prostate volume; *V100* = volume of the prostate receiving 100 % of the prescribed dose

*V150* = volume of the prostate receiving 150 % of the prescribed dose; *UD90* = the minimal dose received by 90 % of the urethral volume

*UD5* = the minimal dose received by 5 % of the urethral volume; *PI* = permanent iodine-125 seed implantation

*PI+EBRT* = PI combined with external beam radiation therapy

### Planning and implant methods

Analysis according to planning methods revealed that, within the PI group, the seed migration incidence was significantly lower in patients receiving preplanning than in those receiving other planning methods (preplanning plus intraoperative planning). According to implantation methods, no significant difference was observed in either the PI or the PI + EBRT group (Table 1).

### Seed migration incidence stratified by methods of chest radiography

**Fig. 2:**
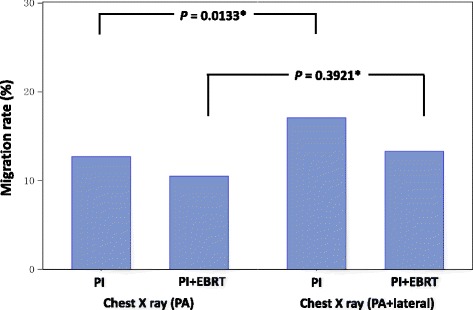
Seed migration rates of each chest X-P methods. PI = permanent iodine-125 seed implantation; PI + EBRT = PI combined with external beam radiation therapy; PA = posterior-anterior *: Chi square test

When patients were divided into those undergoing chest radiography with the PA view alone and those with the PA + lateral views for comparison, the PI group showed that the incidence of seed migration to the lungs was higher in patients undergoing chest radiography with the PA + lateral views than in those undergoing chest radiography with the PA view alone (*p* = 0.0133) (Fig. 2).

### Seed migration incidence stratified by the time of study enrollment

**Fig. 3:**
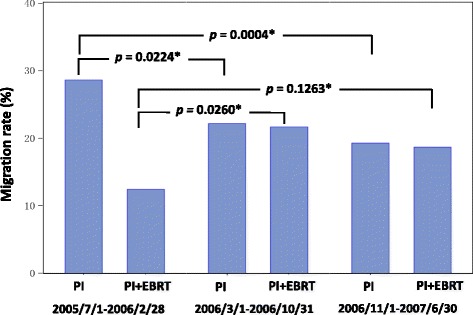
Seed migration rates according to the number of years registered in J-POPS. J-POPS = Japanese Prostate Cancer Outcome Study of Permanent Iodine-125 Seed Implantation; PI = permanent iodine-125 seed implantation; PI + EBRT = PI combined with external beam radiation therapy *: Chi square test

When the seed migration incidence was compared by dividing the two-year study enrollment period into three phases of eight months (early, intermediate, and late), the PI group showed that the incidence was significantly lower in the intermediate and late phases than in the early phase (*p* = 0.0224 and *p* = 0.0004). In the PI + EBRT group, no significant difference was observed (Fig. 3).

### Adverse events

The adverse events associated with the rectum and urination (NCI-CTCAE version 7.0) were divided into those below Grade 2 and those with Grade 2 or above for comparison. During the three years of the follow-up period, neither the PI nor PI + EBRT group showed any significant difference in the grades of adverse events between patients with and without seed migration.

## Discussion

Around the prostate, there is a well-developed venous plexus. It is thought that seed migration occurs because seeds enter the veins and are transported by blood flow [12, 19]. The reported causes for seeds to enter the veins include direct implantation of seeds in the veins [19], an effect of intraabdominal pressure, posture, breathing, gravity, pubic arch interference, oblique needle insertion, number of seeds implanted, number of needles that induces seeds implanted near the veins to enter the veins [9, 18, 24, 28, 29], and edema occurring after implantation [8]. There are many reports describing that, especially when seeds are implanted in the extracapsular area or at the level inferior to the apex of the prostate, the seed migration incidence is high [12, 19]. However, there are also reports that implantation in the extracapsular area does not elevate the seed migration incidence [10, 30].

### Definition of seed migration

Although the transfer of seeds out of the prostate is referred to as seed migration, only the transfer of seeds to the lung is regarded as seed migration in many cases. In the present study, seed migration was defined as “an event in which seeds enter a blood vessel and are transported out of the prostate by blood flow.” Thus, patients in whom seeds were shed into the seminal vesicles or bladder were not included in the group of patients with seed migration. In some of the past reports, movement of seeds within the seminal vesicles and the prostate was also defined as seed migration [20, 31]. Because the definitions vary among reports, the definitions should be checked before making any comparisons of the seed migration incidence.

### Seed migration incidence

The seed migration incidence after PI using loose seeds is reported to range from 1.7 % to 69.4 % [8–13, 19–26] (Table 4). As for the seed migration incidence, there are many reports of studies concerning only migration to the lungs, but only a few reports have included migration to sites other than the lungs [9, 20, 29]. The incidence of seed migration to the lungs alone is reported to be from 1.7–55 %, and that of migration to sites other than the lungs is reported to range from 5.4–7.9 % (Table 4). When migration to the lungs is checked by chest radiography with the PA view alone, it has been reported that migration may be overlooked in 10–15 % of cases [10], and that seeds migrating to the vicinity of the diaphragm may be overlooked [19].

**Table 4:** Seed migration literature review

					Percentage of patients with migration				
Authors	Year	Number of patients	Numbers of PI/PI + EBRT	Isotope	Lung	Others	Lung + Others	Percentage of total seeds	Time of checking for migration
Grimm et al. [21]	1993	221	221/0	I-125/Pd-103 (loose)	17.6	-	-	-	Day 1
Nag et al. [22]	1997	107	107/0	Pd-103 (loose)	17.8	-	-	0.3 (lung)	1 month
Tapen et al. [23]	1998	289	244/45	I-125/Pd-103 (loose, linked)	5.9 (loose 11.0, linked 0.7 )	-	-	-	Day 1
Merrick et al. [10]	2000	175	73/102	I-125/Pd-103 (loose, linked)	21.8 (loose 22.2, linked 21.4)	-	-	0.22 (lung)	<14 days, or >30 days
Older et al. [11]	2001	110	110/0	Pd-103 (loose)	29.0	-	-	-	1-16 months
Ankem et al. [24]	2002	58	Unknown	I-125/Pd-103 (loose)	36.2	-	-	0.72 (lung)	15-90 days (median 45 days)
Eshleman et al. [19]	2004	100	100/0	I-125/Pd-103 (loose)	55.0	-	-	0.98 (lung)	2-3 months
Chauveinc et al. [25]	2004	170	170/0	I-125 (loose)	16.0	-	-	0.26 (lung)	2 months
Kunos et al. [26]	2004	120	120/0	I-125/Pd-103 (loose)	43.0	-	-	0.54 (lung), 0.33 (pelvis)	Median 133 days
Fueller et al. [20]	2004	37	37/0	I-125/Pd-103 (loose)	24.0	5.4	-	0.193 (lung), 0.035 (pelvis)	3-12 months
		23	23/0	I-125/Pd-103 (linked)	0	0	-	0	3-12 months
Stone et al. [12]	2005	238	199/39	I-125/Pd103	1.7	-	-	-	Median 30 months
Saibishkumar et al. [13]	2009	20	20/0	I-125 (loose)	20.0	-	-	0.23	1 month
		20	20/0	I-125 (linked)	0	-	-	0	1 month
Kono et al. [8]	2010	62	62/0	I-125 (loose)	-	-	69.4	0.87 (lung), 1.4 (abdomen + pelvis + thigh)	1 month (scintigraphy)
Sugawara et al. [9]	2011	267	265/2	I-125 (loose)	22.2	7.9	-	0.36 (lung), 0.11 (abdomen + pelvis)	Median 41 months
Taussky et al. [7]	2012	495	495/0	I-125 (loose)	10.3	-	22.8	0.26 (lung), 0.58 (lung + others)	1 month
Miyazawa et al. [29]	2012	121	116/5	I-125 (loose)	-	-	25.6	0.65 (total), 0.28 (lung), 0.34 (others)	12 months
Ishiyama et al. [42]	2014	66	66/0	I-125 (loose)	30.0	39.3 (abdominopelvis)	52*	-	1 month
		74	74/0	I-125 (linked)	0	0	0	-	1 month
J-POPS	2015	1641	1641/0	I-125 (loose)	14.6	11.1	22.7	0.49 (lung+others)	Day 1 to 3 months
		519	0/519	I-125 (loose)	11.2	8.5	18.1	0.53 (lung+others)	Day 1 to 3 months

*: Include lung, abdominopelvis, seminal vesicle and seed positioned more than 1cm from other seeds

*I-125* = idodine-125; Pd-103 = palladium-103; *J-POPS* = Japanese Prostate Cancer Outcome Study of Permanent Iodine-125 Seed Implantation

*PI* = permanent iodine-125 seed implantation; *PI+EBRT* = PI combined with external beam radiation therapy

In our study, the overall seed migration incidence was 21.6 % (PI: 22.7 %, PI + EBRT: 18.1 %); the incidence of seed migration to the lung was 13.8 % (PI: 14.6 %, PI + EBRT: 11.2 %) and the incidence of seed migration to site other than lungs was 10.5 % (PI: 11.1 %, PI + EBRT: 8.5 %). These rates were comparable to those described in previous reports (Table 4). Our study included 1326 patients (61.4 %) who underwent chest radiography with the PA view alone and 834 patients (38.4 %) who underwent chest radiography with the PA + lateral views. The rate of detection of seed migration to the lungs by chest radiography with the PA + lateral views was higher than that by chest radiography with the PA view alone (Fig. 2); in patients undergoing chest radiography with the PA view alone, the incidence of seed migration to the lung might have been underestimated.

Moreover, in cases of seed migration to the heart, chest radiography may not be able to detect migrating seeds because of the heart rate [32]. Thus, CT is reportedly useful for identifying the accurate location of seeds migrating to the chest [14, 33]. However, performing chest CT for confirming migrations may increase the excess radiation exposure. Therefore, performing additional chest CT should be indicated in cases where adverse events occur due to the migrated seeds.

In this study, seed migration to sites other than the lungs was observed in 10.5 % of the patients. In several previous reports, only seed migration to the chest was assessed, whereas few have studied seed migration to sites other than the lungs [9] (Table 4). Seeds may migrate to sites other than the lungs in approximately 5.4–11 % of the cases; thus, not only chest radiography (PA + lateral views) but also abdominal radiography should be performed simultaneously to accurately assess the seed migration incidence after PI [29]. Moreover, there is a report describing that the seed migration sites were confirmed by scintigraphy [8].

In the present study, we divided patients into the PI and PI + EBRT groups for analysis, finding that the seed migration incidence was higher in the PI group than in the PI + EBRT group (Table 1). This is the first report of a comparison between the PI and PI + EBRT groups.

Moreover, within the PI group, the prostate volume and the number of implanted seeds were significantly larger in patients with seed migration (Table 1). Some of the past studies also revealed that the number of implanted seeds is associated with the seed migration incidence [9, 24].

Furthermore, in the present study, the seed migration incidence was significantly lower, as the time of patient enrollment to cohort 1 of the J-POPS study was later (Fig. 3). This is assumed to reflect the effects of the learning curve through the accumulation of experience in the treatment procedure. Taussky et al. have also reported that the seed migration incidence was reduced as the learning curve increased [7].

### Timeline for evaluating seed migration

Because there are cases in which seed migration is not detected on the day of or after PI, but can be confirmed one month later, the seed migration incidence has often been reported to be higher at one month or more after implantation than on the following day [8, 10, 18, 20, 22]. Moreover, migrating seeds that are located in the pelvic cavity on the day after implantation may migrate to the lungs one month later [29]. Based on these reports, if the presence or absence of seed migration is checked only on the day of or after seed implantation, seed migration may be underestimated. Sugawara et al. have reported that the proportions of patients with confirmed seed migration were 13.9 % on the day after implantation, 22.8 % at 14 days after implantation, and 23.6 % at 3 months after implantation, showing an increase over time [18]. When loose seeds are used, the probability of new seed migration occurring one month or more after implantation is extremely low, and it seems appropriate to check seed migration one to three months after implantation instead of immediately after implantation [27]. In the present study, because patients with seed migration that was confirmed one to three months after implantation accounted for 88.6 %, seed migration was evaluated during the appropriate period in the majority of the patients. Although no difference in the seed migration incidence was observed between patients with seed migration confirmed on Day 1 and those with seed migration confirmed at one to three months after implantation, the incidence might have been underestimated in the former cases.

### Adverse events associated with seed migration

The previously reported serious complications of migrating seeds include pneumonia [34], small cell lung cancer [35], myocardial infarction caused by seeds migrating into the coronary arteries [36], and neurological symptoms in the left lower extremity [37]. In the cases of migration into the coronary arteries, it is assumed that a right-to-left atrial or ventricular shunt existed [36]. The present study revealed no difference in adverse events (urination and gastrointestinal symptoms) according to the presence or absence of migrating seeds during the follow-up period, and no adverse events were observed at the sites of seed migration. Although the probability of occurrence of serious complications of seed migration can be expected to be extremely low, careful follow-up observation needs to be continued further in order to determine the presence or absence of an impact at the sites of seed migration.

### Impact on radiation doses

Many of the past reports describe that there was no difference in D90 (Gy) values regardless of the presence or absence of seed migration [9, 23], whereas there are also reports that, with a large number of migrating seeds, V100 (%) and D90 (%) values tended to decrease [38–40]. In the present study, no impact on D90 (Gy) values was observed regardless of the presence or absence of seed migration (Table 3). However, when patients were divided into three groups of those without seed migration, with one migrating seed, and with two or more migrating seeds, the D90 (Gy) and V100 (%) values in the patients with two or more migrating seeds were significantly lower than that in patients without seed migration (Fig. 1). Despite the significant differences in D90 (Gy) values, the differences in the radiation doses were extremely small, and all three groups were exposed, on average, to the prescribed radiation doses or more. Thus, we assume that the impact on the treatment outcomes was small. However, if more seeds migrate, radiation doses will be insufficient, and treatment outcomes may be affected [40]. When insufficient radiation dose due to seed migration is apparent, reimplantation to the target site should be considered [41].

When loose seeds are used, implanting peripheral seeds within the prostate instead of the extraprostatic area is important to reduce seed migration [12, 23].

### Comparison between loose seeds and linked seeds

Methods to reduce seed migration include the implantation of seeds in the capsule and the use of linked seeds. The incidence of migration of linked seeds reportedly ranges from 0 % to 35 %, which is lower than that of loose seeds [13, 23, 38, 42] (Table 4). Because linked seeds could not be used in Japan during the enrollment period of the J-POPS study, the seed migration incidence during this period cannot be compared between loose and linked seeds. In Japan, linked seeds became available in 2013, and since that time, there is a report that the incidence of the migration of linked seeds was lower than that of loose seeds [42]. With regards to radiation dose calculations, some reports describe that D90 (Gy) values were higher for linked seeds than for loose seeds [20, 39, 43], whereas other reports describe that no difference was observed [38, 42, 44, 45]. The opinions are divided. There is also another report that, although differences in D90 (Gy) values were observed between linked and loose seeds, no difference was observed in the seven-year recurrence-free rates based on prostate-specific antigen analysis [46]. Even if radiation doses are affected by the use of linked seeds, the impact on treatment outcomes can be expected to be extremely small. It has been reported that 30 days after treatment with linked seeds, a strand of four linked seeds migrated into the pelvic cavity [28], and that a strand of five linked seeds migrated out from the prostate 30 days after PI [31]. Even linked seeds may migrate into the veins. If linked seeds migrate as a strand, seeds implanted in the tract will migrate out from the prostate at one time, and there may be substantial impact on radiation doses. Moreover, linked seeds implanted in the extracapsular area may migrate when the vicryl sutures or spacers to which the seeds are attached are dissolved and absorbed. Thus, even if seed migration is evaluated on the day of implantation or one to three months after implantation, migration may not have yet occurred. When linked seeds are used, it should be considered to evaluate seed migration at a later time than when loose seeds are used [10, 12]. In clinical practice, when surgeons with techniques to implant loose seeds within the prostate perform implantation, the number of migrating seeds is two seeds or fewer in the majority of the cases, and the impact on D90 (Gy) values is small. However, further long-term follow-up studies are necessary to determine whether seed migration affects treatment outcomes.

### Limitation

In the present multi-institutional study, implantation was usually performed by a different urologist for each facility, and treatment planning was usually conducted by a different radiologist for each facility. Because of the differences among the facilities in terms of the treatment experience of the doctors, radiation dose calculation software, prescribed radiation doses, methods to evaluate seed migration, methods to implant seeds, time of evaluating seed migration, the seed migration incidence varies among the facilities and may be underestimated.

Despite the limitations described above, the results of the present study appear to reflect the current state of the seed migration incidence after treatment with I-125 loose seeds in Japan.

However, because this study had a short follow-up period, long-term follow-up observation is needed in the future to investigate whether adverse events occur at sites of seed migration or how treatment outcomes are affected.

## Conclusions

The seed migration incidence was 21.6 % (PI: 22.7 %, PI + EBRT: 18.1 %), and it was higher in the PI group. In the PI group, the seed migration incidence was higher in those patients with a larger number of implanted seeds. Although there was no difference in D90 (Gy) and V100 (%) values between patients with and without seed migration in the both PI and PI + EBRT groups, the V150 (%), UD90 (Gy), and UD5 (Gy) values were significantly lower in patients with seed migration in the PI group. Thus, when the number of migrating seeds is large, radiation doses may be affected. Moreover, because migration to sites other than the lungs was observed in 10.5 % (PI: 11.1 %, PI + EBRT: 8.5 %), but also abdominal or pelvic radiography as well as chest radiography should be performed to check for seed migration. Additionally, patients who receive PI should be provided with a sufficient explanation of the possible seed migration before treatment.

To the best of our knowledge, this is the first reported prospective cohort study on I-125 loose seed migration, and investigates a record number of cases. It is also the first report to compare the incidence of migration between a PI group and a PI + EBRT group.

## Abbreviations

PI: Permanent iodine-125 seed implantation
EBRT: External beam radiation therapy
PI + EBRT: Permanent seed implantation combined with external beam radiation therapy
I-125: Iodine-125
J-POPS: Japanese Prostate Cancer Outcome Study of Permanent Iodine-125 Seed Implantation
ABS: American Brachytherapy Society
TRI: Translational Research Informatics Center
CT: Computed tomography
CTV: Clinical target volume
PA: Posterior-anterior
NCI-CTCAE: the National Cancer Institute Common Terminology Criteria for Adverse Events
D90: The minimal dose received by 90 % of the prostate volume
UD90: The minimal dose received by 90 % of the urethral volume
UD5: The minimal dose received by 5 % of the urethral volumel
V100: Volume of the prostate receiving 100 % of the prescribed dose
V150: Volume of the prostate receiving 150 % of the prescribed dose
PSA: Prostate specific antigen
BMI: Body mass index
Pd-103: Palladium-103
ADT: Androgen deprivation therapy
